# MCP-induced protein 1 mediates the minocycline-induced neuroprotection against cerebral ischemia/reperfusion injury *in vitro* and *in vivo*

**DOI:** 10.1186/s12974-015-0264-1

**Published:** 2015-02-27

**Authors:** Zhuqing Jin, Jian Liang, Jing Wang, Pappachan E Kolattukudy

**Affiliations:** School of Basic Medicine, Zhejiang Chinese Medical University, Hangzhou, 310053 Zhejiang China; Burnett School of Biomedical Sciences, University of Central Florida College of Medicine, 4000 Central Florida Boulevard, Orlando, FL 32816 USA

**Keywords:** Ischemic stroke, Minocycline, Monocyte chemotactic protein-induced protein 1 (MCPIP1), Middle cerebral artery occlusion (MCAO), Proinflammatory cytokines

## Abstract

**Background:**

Minocycline, a broad-spectrum tetracycline antibiotic, has shown anti-inflammatory and neuroprotective effects in ischemic brain injury. The present study seeks to determine whether monocyte chemotactic protein-induced protein 1 (MCPIP1), a recently identified modulator of inflammatory reactions, is involved in the cerebral neuroprotection conferred by minocycline treatment in the animal model of focal cerebral ischemia and to elucidate the mechanisms of minocycline-induced ischemic brain tolerance.

**Methods:**

Focal cerebral ischemia was induced by middle cerebral artery occlusion (MCAO) for 2 h in male C57BL/6 mice and MCPIP1 knockout mice followed by 24- or 48-h reperfusion. Twelve hours before ischemia or 2 h after MCAO, mice were injected intraperitoneally with 90 mg/kg of minocycline hydrochloride. Thereafter, the animals were injected twice a day, at a dose of 90 mg/kg after ischemia until sacrificed. Transcription and expression of MCPIP1 gene was monitored by quantitative real-time PCR (qRT-PCR), Western blot, and immunohistochemistry. The neurobehavioral scores, infarction volumes, and proinflammatory cytokines in brain and NF-κB signaling were evaluated after ischemia/reperfusion.

**Results:**

MCPIP1 protein and mRNA levels significantly increased in mouse brain undergoing minocycline pretreatment. Minocycline treatment significantly attenuated the infarct volume, neurological deficits, and upregulation of proinflammatory cytokines in the brain of wild type mice after MCAO. MCPIP1-deficient mice failed to evoke minocycline-treatment-induced tolerance compared with that of the control MCPIP1-deficient group without minocycline treatment. Similarly, *in vitro* data showed that minocycline significantly induced the expression of MCPIP1 in primary neuron-glial cells, cortical neurons, and reduced oxygen glucose deprivation (OGD)-induced cell death. The absence of MCPIP1 blocked minocycline-induced protection on neuron-glial cells and cortical neurons treated with OGD.

**Conclusions:**

Our *in vitro* and *in vivo* studies demonstrate that MCPIP1 is an important mediator of minocycline-induced protection from brain ischemia.

## Introduction

Significant advances have been made to lessen the immense public health problem of stroke. Endeavors in prevention have brought down stroke incidence and mortality, and the establishment of exceptional intensive care units has ameliorated the functional consequence of stroke victims [1,2]. However, limited approaches have been made in developing therapies to decrease the detrimental effects of cerebral ischemia. It has been demonstrated that the brain has a marvelous capacity for self-preservation, elucidated by the protective responses induced by ischemia and preconditioning [2]. Understanding the mechanisms underlying these protective actions will help to minimize ischemic brain injury.

Minocycline, a tetracycline antibiotic, manifests anti-inflammatory, anti-apoptotic, and neuroprotective effects independent of its broad-spectrum antimicrobial activity [3-7]. Its superior penetration to the brain tissue, good clinical safety, and prolonged therapeutic window makes it an ideal candidate for application in the treatment of stroke [8]. Although the observed neuroprotection of minocycline in ischemic stroke reflects some properties that mimic selected features of endogenous neuroprotection, it is still unclear whether minocycline acts solely by engaging endogenous neuroprotective mechanisms [2].

Monocyte chemotactic protein-induced protein 1 (MCPIP1) was discovered as an inducible protein expressed in human peripheral blood monocytes treated with monocyte chemotactic protein 1 (MCP-1) [9]. Our previous reports demonstrated that MCPIP1 was a negative regulator of macrophage activation and that MCPIP1 inhibited the production of proinflammatory cytokines, such as TNFα, IL-1β, IL-6, and MCP-1, by inhibiting the activity of JNK and NF-κB proinflammatory signal pathways [10,11]. Our further studies indicated that MCPIP1 was also inducibly expressed in macrophages, endothelial cells, and microglia with LPS stimulation [12-14], and MCPIP1 participates in LPS preconditioning/electroacupuncture-pretreatment-induced ischemic brain tolerance [14,15]. The present study examined whether MCPIP1 is involved in minocycline-treatment-induced neuroprotection against cerebral ischemia/reperfusion injury in neuronal cultures and *in vivo* using MCPIP1-deficient mice.

## Methods

### Animals and minocycline treatment

MCPIP1 knockout mice were established as previously described [11]. Briefly, MCPIP1^−/−^ mice were generated by homologous recombination in embryonic stem cells from C57/BL6 background mice. Exons 3, 4, 5, and most part of 6 of mouse MCPIP1 were replaced with a LacZ-neomycin cassette in embryonic stem cells established from C57/BL6 mice and established MCPIP1^−/−^ mice in pure C57/BL6 background. The absence of MCPIP1 protein in MCPIP^−/−^ mice was confirmed by immunoblotting. In all experiments, 8- to 9-week-old mice were used. All experimental procedures were approved by the Institutional Animal Care and Use Committee of University of Central Florida. We performed all the experiments by using littermate mice. Minocycline treatment was performed following a modification of the method reported by Yrjanheikki and colleagues [3]. Twelve hours before ischemia or 2 h after stroke, mice were injected intraperitoneally with 90 mg/kg of minocycline hydrochloride (Sigma, USA). Thereafter, the animals were injected twice a day, at a dose of 90 mg/kg, after ischemia until sacrificed. Control groups were treated with the same volume of saline starting 12 h before and after stroke.

### Mice focal brain ischemia reperfusion model

For focal brain ischemia, mouse transient middle cerebral artery occlusion (MCAO) was produced by filament occlusion of the right MCA as previously described [14]. In brief, mice were anesthetized with isoflurane (induction with 3%; maintenance with 1.2%) in oxygen-enriched air by facemask, and rectal temperature was controlled at 37°C ± 0.5°C throughout the experiment with heating lamps. Unilateral MCAO was performed by inserting a 7-0 nylon monofilament into the internal carotid artery via an external carotid artery stump and then positioning the filament tip for occlusion at a distance of 8 to 9 mm beyond the internal carotid/pterygopalatine artery bifurcation. MCA was occluded for 120 min followed by reperfusion.

### Brain infarction measurement

The brains were stained with 2,3,5-triphenyltetrazolium chloride (TTC) ( Sigma, USA ) to determine infarct volume [14]. After 2 h of MCAO and 48 h of reperfusion, mice were anesthetized with 4% isoflurane and brains were removed and sectioned coronally at a thickness of 2 mm and incubated in 2% TTC at 37°C for 20 min. Brain slices were then fixed in 4% paraformaldehyde at 4°C overnight and scanned into a computer and quantified using the Image J software. Infarct volume was expressed as a percentage of the contralateral hemisphere. Eight male mice were used for each group.

### Neurological function assessment

The functional outcome of the animals was assessed at 48 h after ischemic/reperfusion. A modified Bederson score [15] was determined according to the following scoring system: 0, no deficit; 1, forelimb flexion; 2, as for 1, plus decreased resistance to lateral push; 3, unidirectional circling; 4, longitudinal spinning or seizure activity; and 5, no movement. Forty-eight hours after surgery, the foot fault test was performed. Ten male mice were used in each group.

#### Brain edema measurement

The mice were anesthetized with 4% isoflurane and brains were removed at 48 h after MCAO. The brains were weighed to obtain the wet weight and were then dried at 105°C for 24 h before measuring dry weight. Brain moisture content (%) was calculated as follows: 100  ×  (wet weight  − dry weight)/wet weight. There were ten mice in each group.

### Cell culture and transfections

Primary culture of mouse cortical neurons was prepared using 18-day-old (E18) embryos from C57 BL/6 J wild type and MCPIP1^−/−^ mouse by the method reported [16] with some modifications. Briefly, the mouse brain cortex was dissected and minced and digested in 0.25% trypsin in HEPES-buffered Hank’s balanced salt solution (HBSS) without calcium or magnesium at 37°C for 10 min. The tissue was washed and dissociated mechanically in HBSS buffer by using a Pasteur pipette. The cell suspension was laid on Hanks’ balanced salt solution containing 4% BSA and centrifuged at 1,000 rpm for 5 min. The pellet was resuspended in Neurobasal-A medium with B-27 supplement, 0.25 mmol/L Glutamax-1, 40 U/mL penicillin, 40 μg/mL streptomycin (Neurobasal-A/B-27), and 10% dialyzed horse serum. Cells were plated at in poly-L-lysine-coated 24-well plates at 6 × 10^5^ cells per well. Cultures were maintained at 37°C in a humidified atmosphere containing 5% CO_2_. Half of the medium was replaced with Neurobasal-A/B-27, 10% fetal bovine serum, and 5% horse serum twice a week. For the oxygen glucose deprivation (OGD), we replaced the growth medium with glucose-free culture medium (2 mL per well) and put the plates into an incubator under 95% N_2_ and 5% CO_2_ at 37°C for 120 min. Then, cells were returned to the normal feeding medium and incubated under normal conditions at 37°C for our studies. Control cell cultures not deprived of oxygen and glucose were incubated under normal conditions. Minocycline (10 μM) was added to the culture 12 h prior to OGD treatment. Referring to the methods in the report [16], transfections of neurons at day 5 *in vitro* (DIV5) with MCPIP1 siRNA (4390771, Life Technologies) and negative control siRNA (4390843, Life Technologies) were performed with Lipofectamine 2000 (Invitrogen) according to the manufacturer’s instructions. Twenty-four hours later, the cells were treated with or without minocycline (10 μM) for 12 h prior OGD treatment.

### MTT and trypan blue exclusion assay to determine cell viability

Primary neuron-glial cells were seeded in 24-well plate. Cells were treated with OGD in the presence or absence of minocycline (10 μM). Twenty-four hours after OGD, examination of cell damage was quantitatively assessed by measuring the reduction of 3-[4,5-dimethylthiazol-2-yl]-2,5-diphenyltetrazolium bromide (MTT) [17] and trypan blue inclusion [18]. MTT (Sigma-Aldrich, USA) was added (final concentration, 0.5 mg/mL) to each well. After 3 h of additional incubation, 100 μL of a solution of 10% sodium dodecyl sulfate (SDS) and 0.01 N HCl was added to dissolve the crystals for 16 h. Absorbance values at the test wavelength of 570 nm and the reference wavelength of 630 nm were determined with an automatic microplate reader. Cell survival was quantitated after trypan blue staining by cell counting. Trypan blue (Sigma, USA) (5%) was added to culture wells, and stained (dead) and unstained (live) cells were counted in five high-power (×200) fields per culture. The extent of cell death was expressed as the number of trypan-blue-positive cells as a percent of the total number of cells counted. At least 200 cells were counted per culture.

### Quantitative real-time PCR

Quantitative real-time PCR was performed as previously described [11]. Briefly, total RNA was isolated using RNA STAT-60 reagent (Tel-Test, Inc., USA) after removing the genomic DNA using DNase I (Ambion, USA), and 2.0 μg of total RNA from microglia or mouse brain tissue was reverse-transcribed to cDNA using a commercially available kit (Applied Biosystems, USA). Quantitative real-time PCR was performed with iCycler Thermal Cycler (Bio-Rad, USA) using 2X SYBR Green master mixes (Bio-Rad, USA). Forty cycles were conducted as follows: 95°C for 30 s, 60°C for 30 s, proceeded by 10 min at 95°C for polymerase activation. Quantification was performed by the delta cycle time method, with mouse or rat β-actin used for normalization. The mouse specific primers (IDT, USA) are as follows: MCPIP1: F: 5′-CCCCCTGACGACCCTTTAG, R: 5′-GGCAGTGGTTTCTTACGAAGGA; TNFα: F: 5′-CTGAGGTCAATCTGCCCAAGTAC, R: 5′-CTTCACAGAGCAATGACTCCAAAG; IL-1β: F: 5′-GCCCATCCTCTGTGACTCAT, R: 5′-AGGCCACAGGTATTTTGTCG; IL-6: F: 5′-TCGTGGAAATGAGAAAAGAGTTG, R: 5′-AGTGCATCATCGTTGTTCATACA; MCP-1: F: 5′-CCATCTCTGACCTGCTCTTCCT, R: -AGACCCACTCATTTGCAGCAT; β-actin: F: 5′-AAATCGTGCGTGACATCAAAGA, R: 5′-GGCCATCTCCTGCTCGAA. The rat specific primers are as follows: TNFα: F: 5′-TCAGCCGATTTGCCATTTC -3′, R: 5′-AGGGCTCTTGATGGCAGAGA-3′; β-actin: F: 5′-GCCTCACTGTCCACCTTCCA-3′, R: 5′-GGGCCGGACTCATCGTACT-3′. Eight male mice were used in each group.

### Immunoblot

Immunoblot was performed as previously described [11]. Proteins from mouse brain tissue were extracted and protein concentrations were determined by the Bradford method (Bio-Rad, USA) with bovine serum albumin as the standard. Proteins (50 μg) were separated by SDS-PAGE and transferred onto nitrocellulose membranes in a transfer buffer containing 0.1% SDS. The membranes were treated with 5% nonfat dry milk in 0.05% Tween 20 in Tris-buffered saline (TTBS) for 2 h and incubated with the primary antibodies against MCPIP1 (Santa Cruz, USA), phosphor-p-65 (Cell Signaling, USA), p-65 (Cell Signaling, USA) at a 1:1,000 dilution in the blocking buffer, 4°C, gently shaking, overnight. After being washed with TTBS three times for 10 min each, the membranes were incubated with a 1:2,000 dilution of secondary antibody (Santa Cruz, USA) in TTBS for 1 h. Following three 10-min washes with TTBS, membranes were incubated with SuperSignal West Pico Chemiluminescent Substrate (Pierce, USA) and exposed to x-ray film. The intensity of bands was quantified by AlphaImage 2200 (AlphaInnotech, USA). The ratios between interested protein bands and the loading control (β-actin, total p-65) were calculated, and the data are expressed as the normalized folds with respect to sham. Six male mice were used in each group.

### Immunohistochemistry

Immunohistochemistry was performed as previously described [15]. At 24 h after minocycline treatment, mice were transcardially perfused under anesthesia with ice-cold phosphate-buffered saline and then with 4% paraformaldehyde. Brains were removed and fixed overnight in 4% paraformaldehyde at 4°C. The brains were sectioned coronally at 30-μm thickness in ice-cold phosphate-buffered saline using a vibrating microtome (Leica Microsystems). The sections were placed in an anti-freeze solution and stored at −20°C for later use. They were washed, the nonspecific binding sites on sections were blocked with 3% bovine serum albumin, and incubated overnight with primary antibody against glial fibrillary acidic protein (GFAP), primary antibody against CD11b (BD Biosciences Pharmingen, San Diego, CA, USA), primary antibody against neuron-specific enolase (NSE; Invitrogen, USA), primary antibody against MCPIP1 (Santa Cruz Biotechnology, Dallas, Texas, USA), AlexaFluor®-488-conjugated secondary antibody (Invitrogen, USA), and AlexaFluor®-594-conjugated secondary antibody (Invitrogen, USA) and scanned under a fluorescence microscope (Leica TCS SP5) at × 400 magnification. Three fields per section were captured and analyzed. Six male mice were used in each group.

### Statistical analysis

The data are presented as mean ± SD. Multiple comparisons were evaluated by one-way ANOVA followed by the Tukey or Dunnett test. Two-group comparisons were analyzed by the two-tailed Student *t* test. For all analyses, a value of *P* < 0.05 was considered significant.

## Results

### MCPIP1 induction in mouse brain by minocycline

**Figure 1:**
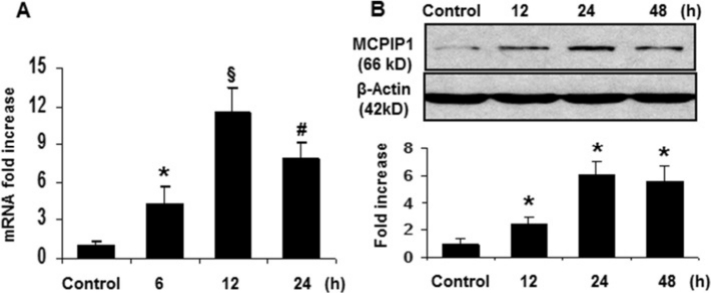
**Minocycline-treatment-induced MCPIP1 in the brain. (A)** MCPIP1 mRNA expression in mouse brain by minocycline treatment as measured by qRT-PCR. Values represent mean ± SD, *P < 0.05, ^#^ *P* < 0.01, ^§^ *P* < 0.001 versus control. **(B)** MCPIP1 protein levels in mouse brain by minocycline treatment as measured by Western blot. Results are representative of three independent experiments. *P < 0.05 versus control. Values represent mean ± SD. MCPIP1, monocyte chemotactic protein-induced protein 1; qRT-PCR, quantitative real-time PCR.

**Figure 2:**
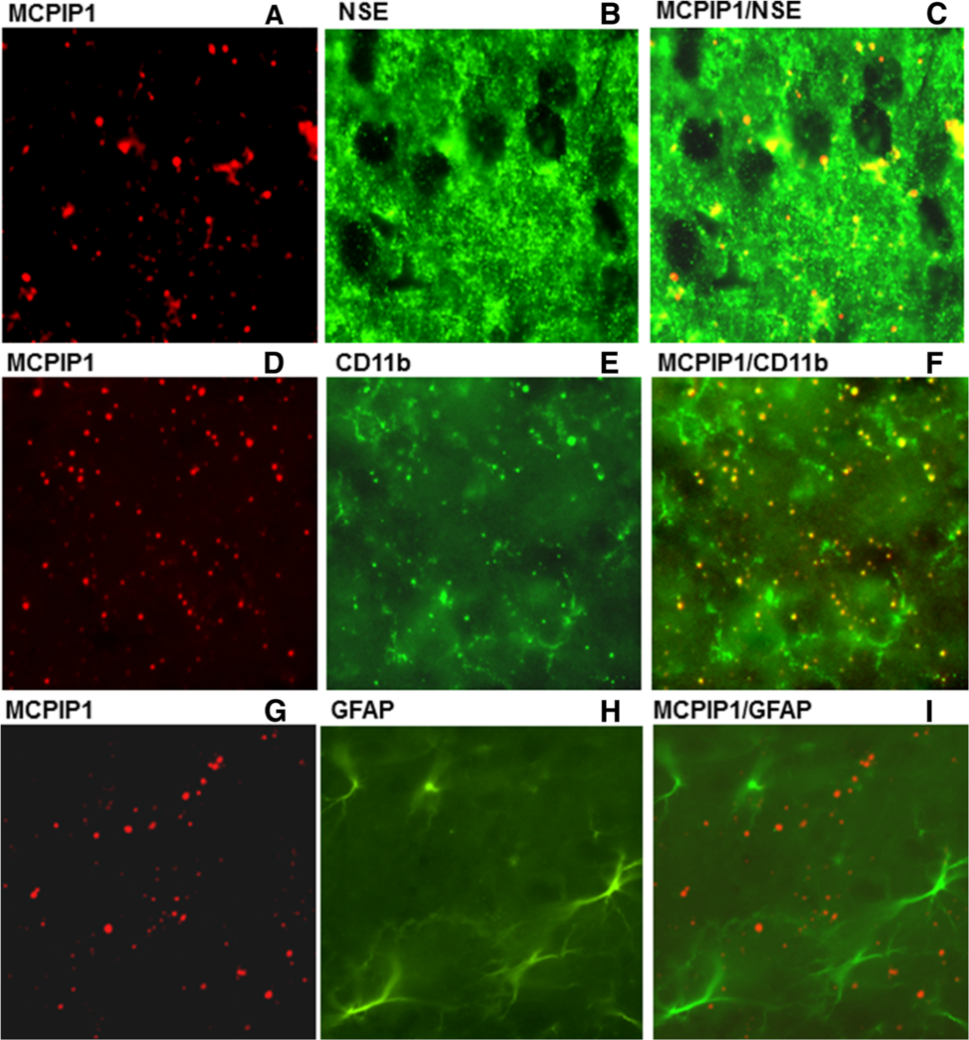
**MCPIP1 is expressed in neurons and microglia.** Co-localization of MCPIP1 expression **(A, D, G)** of NSE **(B)**, CD11b **(E)**, and GFAP **(H)** at 24 h after minocycline treatment. Yellow fluorescence indicates co-localization of MCPIP1/NSE **(C)** and MCPIP1/CD11b **(F)**. MCPIP1/GFAP **(I)** showed no yellow-fluorescent structures. *n* = 5 mice per group. GFAP, glial fibrillary acidic protein; MCPIP1, monocyte chemotactic protein-induced protein 1; NSE, neuron-specific enolase.

In ischemic stroke, minocycline treatment has been demonstrated to have a significant protective effect against brain damage, but the underlying mechanism remains poorly understood. First, we examined whether minocycline treatment induces MCPIP1 in mouse brain. The MCPIP1 mRNA level in mouse cortex brain was significantly induced by minocycline treatment compared to controls. Significant increase of MCPIP1 transcript level was detected at 6 h and reached 11.5 ± 1.9-fold at 12 h after minocycline treatment (*P* < 0.001) and began to decline by 24 hr (Figure 1A). Consistently, the MCPIP1 protein level in mouse brain was significantly elevated by minocycline treatment than in the controls: 6.1 ± 0.93-fold increase at 24 h after minocycline treatment and remained high till 48 hr (*P* < 0.05; Figure 1B). To determine the cellular localization of MCPIP1 expression, sections from mice brain at 24 h after minocycline treatment were subjected to immunohistochemistry with antibody against MCPIP1. MCPIP1 expression was elevated by minocycline treatment and the immunoreactivity co-localized with NSE, a neuron specific marker, implying that the elevated level of MCPIP1 protein induced by minocycline was located mainly in neurons (Figure 2C). Examination of the co-localization of MCPIP1 with CD11b, the leukocyte-specific receptor and regarded as a marker for macrophages/microglia, showed a few MCPIP1/CD11b-positive cells (Figure 2F). Examination of the co-localization study of MCPIP1 with GFAP, a marker for activated astrocytes, showed that MCPIP1 expression was not located in the astrocytes (Figure 2I). These observations indicate that MCPIP1 protein is upregulated mainly in neurons and microglia in the mice brain after minocycline treatment.

### Loss of minocycline-treatment-induced tolerance to ischemic stroke by MCPIP1 deficiency

**Figure 3:**
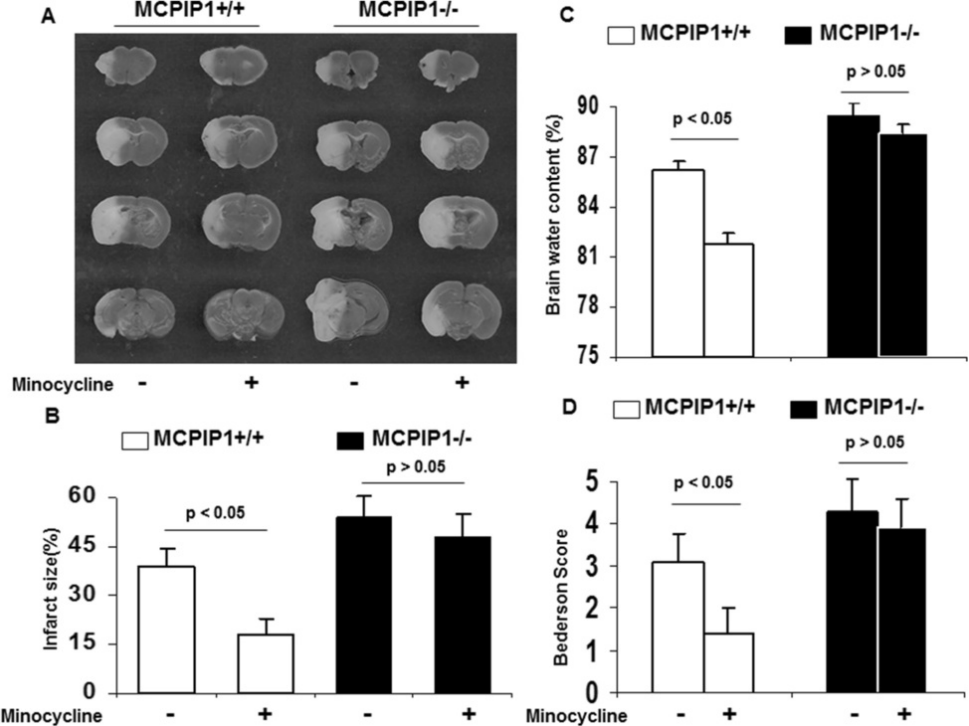
**Reduction in infarct size and improvement of neurological function by minocycline treatment in the wild type, but not in MCPIP1-deficient mice.** The brain infarct size was assessed 48 h after MCAO. **(A)** Infarct images obtained by TTC staining at 48 h after MCAO. The normal tissue was stained deep red and the infarct was stained milky. **(B)** Brain infarcts were quantified as percentage area of ischemic hemisphere. The infarct size of minocycline-pretreated wild type mice was significantly reduced compared to that of the control. There was no significant difference in brain infarct size between the minocycline-pretreated and control in MCPIP1-deficient mice. **(C)** Brain water content as a measure of brain edema of the ischemic hemisphere. The brain edema was significantly reduced at 48 h after MCAO in minocycline-pretreated wild type mice compared to that of the control group. In the MCPIP1-deficient mice, there was no significant difference in brain water content between minocycline-pretreated and control group without minocycline treatment. **(D)** Neurological function assessment was performed 24 h after MCAO. The neurological scores of minocycline-pretreated wild type mice were significantly improved compared to that of the control. In MCPIP1-deficient mice, there was no significant difference in neurological deficits between minocycline-pretreated and control group. Values represent mean ± SD, *P < 0.05, *n* = 10 mice per group. MCAO, middle cerebral artery occlusion; MCPIP1, monocyte chemotactic protein-induced protein 1; TTC, 2,3,5-triphenyltetrazolium chloride.

**Figure 4:**
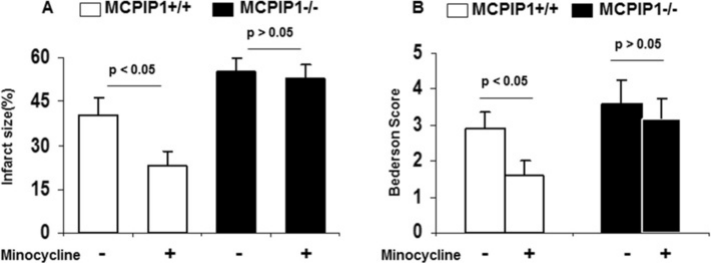
**Reduction in infarct size and improvement of neurological function by 2-h post-treatment of minocycline on stroke in the wild type, but not in MCPIP1-deficient mice. (A)** The results showed that the infarct size of minocycline-treated wild type mice was significantly reduced compared to that of the control (40.3% ± 6.1% versus 23.3% ± 4.7%, respectively; values represent mean ± SD, *P < 0.05, *n* = 10 mice per group). MCPIP1-deficient mice failed to evoke minocycline treatment-induced neuroprotection compared with that of control MCPIP1 knockout group without minocycline treatment (55.1% ± 4.9% versus 52.8% ± 5.3%). There was no significant difference in brain infarct size between minocycline-treated and control in MCPIP1 knockout mice. **(B)** The neurological functions of mice were determined, and the results showed that the neurological scores of minocycline-treated wild type mice were significantly improved compared to that of the control. In MCPIP1-deficient mice, there was no significant difference in neurological deficits between minocycline-treated and control group without minocycline treatment. MCPIP1, monocyte chemotactic protein-induced protein 1.

We examined the effects of minocycline treatment on ischemic brain infarction. MCPIP1-deficient and wild type mice were pretreated with minocycline as described in the Methods section, and these mice were subjected to MCAO 12 h after minocycline treatment. The brain infarct size was assessed with TTC staining 48 h after MCAO, and the results showed that the infarct size of minocycline-pretreated wild type mice was significantly reduced compared to that of the control (39.4% ± 5.3% versus 19.3% ± 4.1%, respectively, Figure 3A). MCPIP1-deficient mice failed to evoke minocycline-treatment-induced tolerance compared with that of the control MCPIP1 knockout group without minocycline treatment (54.3% ± 6.5% versus 47.6% ± 6.9%, respectively, Figure 3A). There was no significant difference in brain infarct size between minocycline-pretreated and control in MCPIP1 knockout mice. The neurological functions of mice were determined, and the results showed that the neurological scores of minocycline-pretreated wild type mice were significantly improved compared to that of the control. In MCPIP1-deficient mice, there was no significant difference in neurological deficits between the minocycline-pretreated and control group without minocycline treatment (Figure 3B). Edema is one of the earliest pathological changes after ischemic neuronal damage. The results showed that the brain edema was significantly reduced at 48 h after MCAO in minocycline-pretreated wild type mice compared to that of the control group. In the MCPIP1-deficient mice, there was no significant difference in brain water content between the minocycline-pretreated and control group without minocycline treatment (Figure 3C). We also examined the effects of minocycline treatment post-stroke on ischemic brain infarction and neurological functions. MCPIP1-deficient and wild type mice were treated with minocycline (50 mg/kg per day) at 2 h after MCAO. The results showed that the infarct size of minocycline-treated wild type mice was significantly reduced compared to that of the control (40.3% ± 6.1% versus 23.3% ± 4.7%, *P* < 0.05, Figure 4A). MCPIP1-deficient mice failed to evoke minocycline-treatment-induced neuroprotection compared with that of the control MCPIP1 knockout group without minocycline treatment (55.1% ± 4.9% versus 52.8% ± 5.3%, *P* > 0.05, Figure 4A). There was no significant difference in brain infarct size between the minocycline-treated and control in MCPIP1 knockout mice. The neurological functions of mice were determined, and the results showed that the neurological scores of minocycline-treated wild type mice were significantly improved compared to that of the control. In MCPIP1-deficient mice, there was no significant difference in neurological deficits between the minocycline-treated and control group without minocycline treatment (Figure 4B). These results indicated that MCPIP1 may mediate minocycline-induced neuroprotection afforded by minocycline administration after stroke or before stroke.

### Proinflammatory cytokine expression

**Figure 5:**
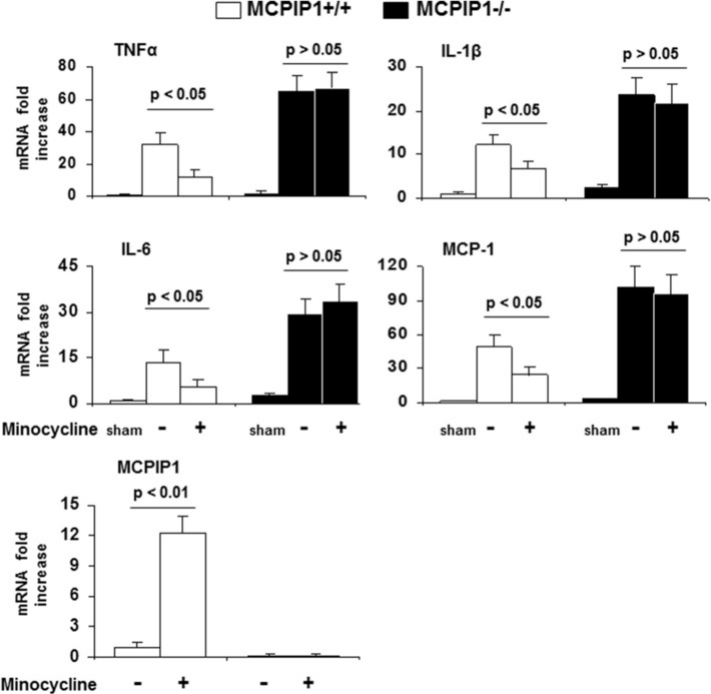
**Reduction in inflammatory cytokine expression in ischemic brain by minocycline treatment in the wild type, but not in MCPIP1-deficient mice.** The results showed that the expression levels of TNFα, IL-1β, IL-6, and MCP-1 were significantly elevated in shams in MCPIP1-deficient mice than that of wild type and that the expression levels of TNFα, IL-1β, IL-6, and MCP-1 were significantly reduced at 24 h after MCAO in minocycline-pretreated wild type mice compared to that of the control. In the MCPIP1-deficient mice, there was no significant difference in proinflammatory cytokine expression between minocycline-pretreated and control group without minocycline treatment. The MCPIP1 mRNA level in mouse brain was significantly induced by minocycline treatment compared to controls in wild type mice. Significant increase of MCPIP1 transcript level was detected at 24 h after minocycline treatment after ischemic stroke (*P < 0.01*). Values represent mean ± SD, *n* = 6 mice per group. MCPIP1, monocyte chemotactic protein-induced protein 1; MCP-1, monocyte chemotactic protein 1.

We examined the expression of proinflammatory cytokine transcripts in the ischemic brain of the wild type and MCPIP1 knockout mice with or without minocycline treatment after MCAO. The results showed that the transcript levels of TNFα, IL-1β, IL-6, and MCP-1 were significantly elevated in shams in MCPIP1 knockout mice than that of wild type. The transcript levels of TNFα, IL-1β, IL-6, and MCP-1 were significantly reduced at 24 h after MCAO in minocycline-pretreated wild type mice compared to that of the control group. In the MCPIP1-deficient mice, there was no significant difference in the proinflammatory cytokine expression between the minocycline-pretreated and control group without minocycline treatment (Figure 5). Consistent with the results of Figure 1, the MCPIP1 mRNA level in mouse brain was significantly induced by minocycline treatment compared to the controls in wild type mice. Significant increase of the MCPIP1 transcript level was detected at 24 h after minocycline treatment after ischemic stroke (Figure 5).

### Minocycline protection from OGD-induced neuronal damage via induction of MCPIP1 in neurons

**Figure 6:**
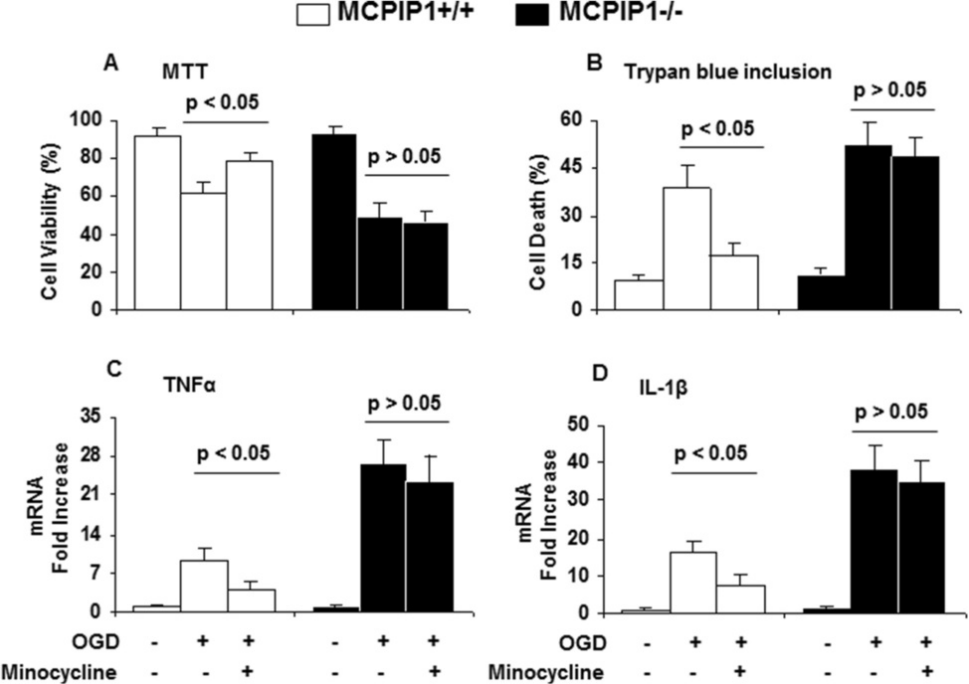
**Minocycline neuroprotection to OGD-induced neuronal damage via MCPIP1 in neuron-glia cells from MCPIP1** ^**−/−**^ **mice. (A, B)** Cell viability assays by MTT and cell death rate by trypan blue inclusion revealed that treatment of OGD on the mixed neuron-glia cells resulted in cell death and pretreatment with minocycline can increase the resistance to OGD-induced neuronal damage. Conversely, there was no significant difference on OGD-induced neuronal damage in neuron-glia cells from MCPIP1^−/−^ mice with the treatment of minocycline compared with that of the control group without minocycline treatment. **(C, D)** Pretreatment of minocycline decreased OGD-induced expression of TNFα and IL-1β in the mixed neuron-glia cells. There was no significant reduction of OGD-induced TNFα and IL-1β expression in mixed neuron-glia cells from MCPIP1^−/−^ mice with the treatment of minocycline compared with that of the control group without minocycline treatment. MCPIP1, monocyte chemotactic protein-induced protein 1; MTT, 3-[4,5-dimethylthiazol-2-yl]-2,5-diphenyltetrazolium bromide; OGD, oxygen glucose deprivation.

**Figure 7:**
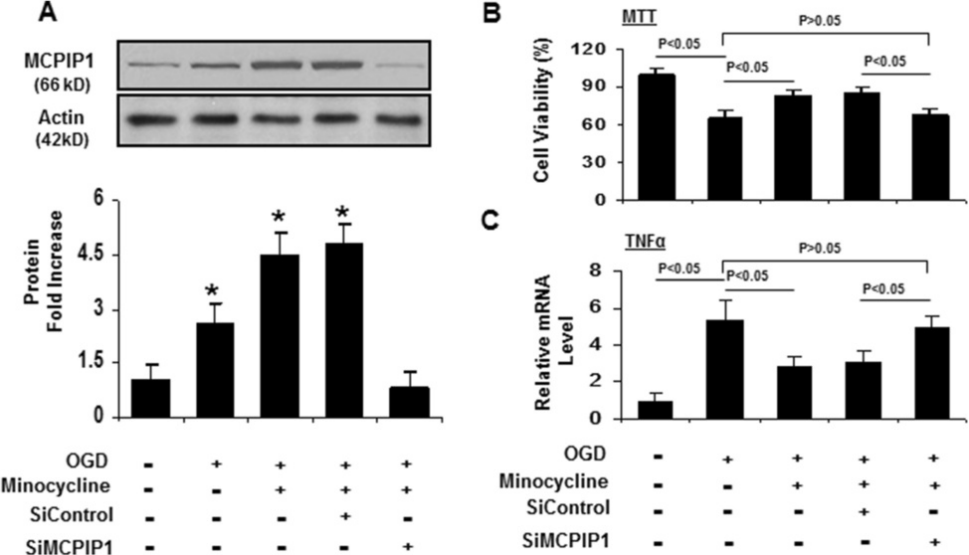
**Minocycline neuroprotection to OGD-induced neuronal damage via MCPIP1 in primary cortical neurons from wild type mice. (A)** A representative Western blot shows the induced expression of MCPIP1 by minocycline and the knockdown of MCPIP1 by siMCPIP1 in primary cortical neurons from C57BL/6 wild type mice. OGD induced MCPIP1 mildly, and minocycline induced MCPIP1 to a much higher level. siMCPIP1 knocked down MCPIP1 very efficiently. **(B)** MTT cell viability analysis shows that pretreatment with minocycline increased the resistance to OGD-induced neuronal damage and that knockdown of MCPIP1 by siMCPIP1 reduced the protective effects of minocycline on primary cortical neurons subjected to OGD. **(C)** Knockdown of MCPIP1 significantly increased the expression of TNFα induced by OGD in primary cortical neurons pretreated with minocycline compared with that of control group of siControl. MCPIP1, monocyte chemotactic protein-induced protein 1; MTT, 3-[4,5-dimethylthiazol-2-yl]-2,5-diphenyltetrazolium bromide; OGD, oxygen glucose deprivation; siControl, negative control silencing RNA; siMCPIP1, MCPIP1 silencing RNA.

Given the observed effects of minocycline on wild type and MCPIP1^−/−^ mice subjected to MCAO, we investigated the roles of minocycline on OGD-induced neuronal damage in primary neuron-glia cells from wild type and MCPIP1^−/−^ mice. Cell viability assays revealed that the treatment of OGD on the mixed neuron-glia cells resulted in cell death, and pretreatment with minocycline (10 μM) increased the resistance to OGD-induced neuronal damage (Figure 6A, B) and reduced the expression of TNFα and IL-1β induced by OGD in neuron-glia cells (Figure 6C, D). More significantly, cell viability analysis revealed that depletion of MCPIP1 in neuron-glia cells from MCPIP1^−/−^ mice reduced the protective effects of minocycline on the cells subjected to OGD. In addition, MCPIP1 depletion in neuron-glia cells also increased the expression of TNFα and IL-1β induced by OGD in the cells pretreated by minocycline compared with that of the control group from the wild type. We also investigated the roles of minocycline on OGD-induced neuronal damage in primary cortical neurons from wild type mice with or without knockdown of MCPIP1. Minocycline (10 μM) significantly induced the expression of MCPIP1 in cortical neurons (Figure 7A). Cell viability assays revealed that the treatment of OGD on cortical neurons resulted in cell death, and the pretreatment with minocycline can increase the resistance to OGD-induced neuronal damage (Figure 7B) and reduced the expression of TNFα induced by OGD in cortical neurons (Figure 7C). More interestingly, cell viability analysis revealed that the depletion of MCPIP1 by siMCPIP1 reduced the protective effects of minocycline on cortical neurons subjected to OGD (Figure 7B). In addition, MCPIP1 depletion significantly increased the expression of TNFα induced by OGD in cortical neurons pretreated by minocycline compared with that of the control group of siControl (Figure 7C).

### Activation of NF-κB-signaling pathway

**Figure 8:**
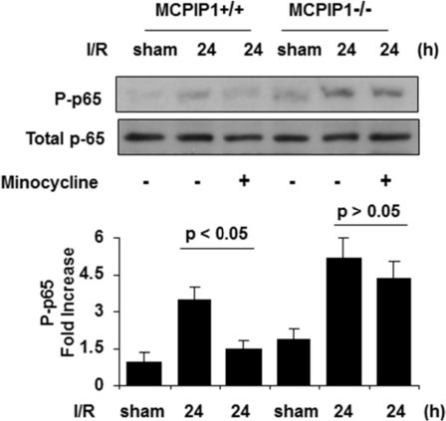
**Inhibition of NF-κB activation in the ischemic brain by minocycline treatment in the wild type, but not in MCPIP1-deficient mice.** A representative Western blot shows protein levels of p-65 phosphorylation. The phosphorylation of p-65 was significantly reduced at 24 h after MCAO in minocycline-pretreated wild type mice compared to that of the control. In MCPIP1-deficient mice, there was no significant difference in p-65 phosphorylation level between the minocycline-pretreated and control group without minocycline treatment. Densitometric analysis was used to quantify phospho-p-65 protein levels versus total p-65 in three independent Western blots, and the data are expressed as the normalized folds with respect to sham. Values represent mean ± SD. MCAO, middle cerebral artery occlusion; MCPIP1, monocyte chemotactic protein-induced protein 1.

Since activation of the NF-κB-signaling pathway is involved in the production of proinflammatory cytokines, we tested whether minocycline treatment affects NF-κB activation. The results showed that phosphorylation of p-65 was significantly reduced at 24 h after MCAO in the minocycline-pretreated wild type mice compared to that of the control. In MCPIP1-deficient mice, there was no significant difference in p-65 phosphorylation level between the minocycline-pretreated and control group without minocycline treatment (Figure 8).

## Discussion

Many studies have shown that doxycycline and minocycline play anti-inflammatory and neuroprotective effects in ischemic brain injury. This action is completely independent and distinct from their antimicrobial action [3,8,19-22]. It has been well known that ischemic stroke includes secondary inflammation that plays a significant part in the devastating consequences of ischemic insult [23-27]. Although numerous studies have shown that minocycline can provide significant neuroprotection against ischemic brain injury and inhibit inflammatory responses such as microglial activation and proinflammatory cytokine generation [3,27-29], the molecular mechanisms that contribute to the alleviation of brain inflammation after ischemia by minocycline are not well understood. The present study is the first to reveal the role of MCPIP1 in minocycline-treatment-induced cerebral ischemic protection. MCPIP1 was first identified as a protein induced in human peripheral blood monocyte by the chemokine MCP-1 [9]. MCPIP1 has been demonstrated to have the ability to suppress inflammation [10,11,30]. MCPIP1 negatively affects inflammatory gene expression and NF-κB activation in response to LPS [10,11]. In our previous studies, MCPIP1 was also found to be inducibly expressed in macrophages, macroglia, and endothelial cells with LPS stimulation [12-14]. It was also shown that MCPIP1 participates in LPS and electroacupuncture preconditioning-induced ischemic brain tolerance [14]. The recent study [31] showed that MCPIP1 can be translationally controlled by some other proinflammatory cytokines such as IL-17. All of these investigations indicated that MCPIP1 may act as an inducible endogenous negative feedback regulator of inflammation and could play a highly beneficial role in human inflammation-related diseases including stroke. In the present study, we found that MCPIP1 was significantly induced in mouse brain by minocycline treatment, which suggested that MCPIP1 may be involved in minocycline-induced neuroprotection after ischemic stroke. In addition, we observed that brain infarct volumes, brain edema, and neurological functions after stroke were significantly alleviated by minocycline treatment in wild type mice, whereas in MCPIP1-deficient mice, minocycline treatment failed to improve these outcomes of ischemia, which indicated that MCPIP1 may participate in minocycline-induced neuroprotection in ischemic stroke. Many studies have demonstrated that minocycline is an ideal therapeutic agent for stroke. Our results show that administration of minocycline before or after stroke afforded protection against stroke damage. Of course, the post-treatment paradigm of minocycline indicates more clinical significance in its potential therapeutic application for stroke in the future. The novelty of the present study is that MCPIP1 mediates minocycline-induced neuroprotection against cerebral ischemia/reperfusion injury whether the drug is ministered before or after stroke. In both cases, minocycline induced MCPIP1 in the brain and afforded protection against ischemic brain damage.

It has been well established that proinflammatory gene expression leads to stroke damage [25,32]. During brain ischemia, proinflammatory cytokines such as TNF-α, IL-1β, IL-6, and chemokines such as CINC and MCP-1 are produced by a variety of activated cell types, including endothelial cells, microglia, astrocytes, and neurons [32]. Blocking the production of these proinflammatory cytokines would be an important strategy to protect against ischemia brain injury. In this study, we observed that TNFα, IL-1β, IL-6, and MCP-1 expressions were significantly reduced at 24 h after MCAO by minocycline treatment. In MCPIP1-deficient mice, minocycline treatment failed to suppress the production of such cytokine. Furthermore, we found that expression of MCPIP1 protein was upregulated by minocycline treatment mainly in neurons and microglia, which are the main source of proinflammatory cytokines during ischemia. These results implied that MCPIP1 induction by minocycline may play a beneficial role by inhibiting the generation of proinflammatory cytokines in neurons and microglia during brain ischemia. Activation of NF-κB-signaling pathways leads to inflammatory cytokine production [11]. In our previous study, we found that MCPIP1 can also act as a deubiquitinase to negatively regulate NF-κB signaling by targeting TNF receptor-associated factors (TRAFs) [11,33]. In the present study, we found that phosphorylation of p-65 was significantly reduced at 24 h after MCAO in minocycline-treated wild type mice compared to that of the control. In MCPIP1-deficient mice, there was no significant difference on p-65 phosphorylation level between the minocycline-treated and control group without minocycline treatment. Our study suggested that enhanced activation of NF-κB-signaling pathway in MCPIP1-deficient mice leads to increased proinflammatory cytokine production after brain ischemia with minocycline treatment and that MCPIP1 is involved in minocycline-treatment-induced inhibition of the NF-κB-signaling pathway after ischemic stroke. In the present study, minocycline treatment showed significant neuroprotection in mice subjected to focal brain ischemia by MCAO, which is consistent with other reports. However, we found there was significant loss of minocycline-treatment-induced ischemic brain tolerance in MCPIP1-deficient mice after ischemic stroke, which indicated that MCPIP1 may mediate the beneficial role in minocycline-treatment-induced neuroprotection after focal brain ischemia.

It is possible that the absence of MCPIP1 in the mice creates systemic inflammation that minocycline treatment cannot overcome. However, our *in vitro* studies in mixed neuron-glia cells and primary cortical neurons indicated that minocycline-pretreatment-induced neuroprotection is, at least partly, via MCPIP1, as absence of MCPIP1 in mixed neuron-glia cells and knockdown of MCPIP1 in primary cortical neurons with OGD treatment resulted in a loss of minocycline-induced protection, which could largely exclude the potential affects of systemic consequence resulting from *in vivo* studies of general knockout mice. Considering the other experimental results in this study, including that MCPIP1 can be significantly induced by minocycline treatment in the brain, and that MCPIP1 has been identified as an important inducible anti-inflammatory regulator in stroke pathophysiology [11], it would appear more likely that MCPIP1 actually participates in the minocycline-treatment-induced neuroprotection. Mice with brain-specific MCPIP1 deficiency might provide a suitable model to explore the role of MCPIP1 in ischemic-stroke-induced brain damage without the complication caused by the general knockout of MCPIP1. Based on our *in vivo* and *in vitro* studies, we may conclude that MCPIP1 induction is involved in minocycline-induced neuroprotection from ischemic brain injury.

All these findings indicate that the endogenous inducible MCPIP1 protein may serve as an important common shared mediator which performs negative feedback to reduce the inflammatory responses caused by a wide range of hazardous stresses.
